# A first analysis of excess mortality in Switzerland in 2020

**DOI:** 10.1371/journal.pone.0253505

**Published:** 2021-06-17

**Authors:** Isabella Locatelli, Valentin Rousson

**Affiliations:** Center for Primary Care and Public Health (Unisanté), University of Lausanne, Lausanne, Switzerland; University of South Florida, UNITED STATES

## Abstract

**Objective:**

To quantify excess all-cause mortality in Switzerland in 2020, a key indicator for assessing direct and indirect consequences of the COVID-19 pandemic.

**Methods:**

Using official data on deaths in Switzerland, all-cause mortality in 2020 was compared with that of previous years using directly standardized mortality rates, age- and sex-specific mortality rates, and life expectancy.

**Results:**

The standardized mortality rate was 8.8% higher in 2020 than in 2019, returning to the level observed 5–6 years before, around the year 2015. This increase was greater for men (10.6%) than for women (7.2%) and was statistically significant only for men over 70 years of age, and for women over 75 years of age. The decrease in life expectancy in 2020 compared to 2019 was 0.7%, with a loss of 9.7 months for men and 5.3 months for women.

**Conclusions:**

There was an excess mortality in Switzerland in 2020, linked to the COVID-19 pandemic. However, as this excess only concerned the elderly, the resulting loss of life expectancy was restricted to a few months, bringing the mortality level back to 2015.

## Introduction

The year 2020 will be remembered as the year of the global spread of the COVID-19 pandemic caused by the severe acute respiratory syndrome coronavirus 2 (SARS-CoV-2). By early 2021, more than 150 million cases were confirmed worldwide and more than 3 million deaths were attributed to COVID-19. At a crossroads in the heart of Europe, after a first death recorded on March 5, 2020, Switzerland was particularly affected by the pandemic with more than 650’000 cases and 10’500 deaths attributed to COVID-19 (about 7400 in 2020) for a population of 8.6 million. In 2020, Switzerland had the 21st highest mortality rate due to COVID-19 among 156 countries with a population of at least one million (https://www.worldometers.info/coronavirus/).

Various restrictive and protective measures have been taken around the world, including in Switzerland, to fight the pandemic. The costs in terms of social burdens and economic consequences and benefits in terms of health of these measures are difficult to quantify and will be the subject of long discussions for many years. For such assessments, one key indicator is all-cause mortality. Unlike the specific mortality attributed to COVID-19, which may depend in part on unreliable diagnoses, all-cause mortality is a robust indicator that allows the direct and indirect effects of a pandemic to be taken into account [1].

In this paper, we propose an analysis of all-cause mortality in Switzerland for the year 2020, with the aim of quantifying the excess mortality observed in 2020 compared to previous years in one of the countries with the highest life expectancy in the world (second behind Japan in 2015 according to the World Health Organization [2]). In Switzerland, the Federal Statistical Office (FSO) has been updating weekly the number of deaths by sex and age group for each of the 53 weeks of the year 2020, making these figures available to the public. Since it usually takes a few years to obtain complete and consolidated statistics on deaths in a country, our analysis is referred to as a “first analysis”. This is also the reason why relatively few such analyses have been fully published so far worldwide for the whole year 2020. For example, Aburto et al. [3] studied all-cause mortality in England and Wales, while Woolf et al. [4] and Ahmad et al. [5] did so in the United States, although only the latter considered the entire year 2020. Andrasfay and Goldman [6] provided an estimate of the reduction in life expectancy in the United States in 2020 based on COVID-19 mortality. Heuveline and Tzen [7] applied a similar procedure for 186 countries or territories, including Switzerland. In a preprint, Aburto et al. [8] provided such estimates for 29 countries, including Switzerland, based on all-cause mortality. Given the potential importance of such analyses in the current (urgent) scientific and political debates on the COVID-19 pandemic, we believe that even results that are not entirely definitive deserve to be presented and published.

When analyzing and comparing mortality over the years, a simple look at the total number of deaths is usually misleading because of the tendency of the population to increase, which naturally implies an increasing number of deaths. Therefore, the number of deaths must be divided by the population size, resulting in a so-called crude mortality rate. However, the latter still does not account for changes in the age (and possibly sex) distribution of the population over time [9]. Indeed, the age structure of the European and Swiss population changes each year, moving towards an increasingly older population. As the population ages, crude mortality rates can stay constant or continue to increase even as age-specific mortality decreases.

To overcome this issue, standardized (or adjusted) mortality rates were developed in the first half of the 19th century [10] as a summary measure of mortality that controls for the changes in the age and sex distribution of a population: the age- and sex-specific mortality rates of a study population are applied to the age and sex distribution of a standard population to allow comparisons across countries or time periods [11, 12, Chapter 19 of 13].

To quantify the excess mortality observed in Switzerland in the year 2020, we therefore calculate and compare standardized mortality rates over the years using the Swiss population at the beginning of 2020 as the standard. We then make similar comparisons in selected age groups, separately for men and women, to identify specific age groups most affected by excess mortality. Finally, we also calculate and compare the life expectancies that would result from the age- and sex-specific mortality rates observed over the years, placing the life expectancy of 2020 in a secular perspective.

## Data

We used official data on deaths in Switzerland by sex and age group published for 2020 and previous years (going back to 1970) by the Swiss Federal Statistical Office, FSO (https://www.bfs.admin.ch/bfs/fr/home/statistiques/population/naissances-deces/deces.html). Our last access to this site was on May 19, 2021. The number of deaths, separately for men and women, was available by 1-year age groups (with a last open class of 110+) until 2019, and by 5-year age groups (with a last open class of 90+) for the year 2020, for which the number of deaths is provided for each week. By convention, the year 2020 is divided into 53 weeks, the first of which begins on December 30, 2019 and the last of which ends on January 3, 2021. We therefore corrected the number of deaths in these two weeks that spill over into two other years by excluding 2/7 of the deaths in the first and 3/7 in the last. For leap years (including 2020), we further excluded 1/366 of the deaths.

The size of the Swiss population as of January 1, 2011–2020, stratified by sex and by one-year age groups (with a last open class of 100+), can be found in the FSO database (https://www.bfs.admin.ch/bfs/fr/home/statistiques/population/effectif-evolution.html) and for January 1, 1876–2019, in the Human Mortality Database, HMD (https://www.mortality.org/). Since the differences for the overlapping years are negligible, we used the HMD source for the years 1970–2010 and the FSO source for the years 2011–2020. Life expectancy for both sexes is also available in the HMD database for the period 1876–2018.

## Methods

Directly standardized mortality rates (dSMR) were used to compare mortality in 2020 with mortality in previous years, always considering 2020 as the standard. Algebraically, a dSMR is an average of the age- and sex-specific mortality rates observed in a given year, weighted by the age and sex distribution of the standard population. The dSMR for year *y* (*y* = 1970,….2019) is therefore defined as follows:

$$m^{y}=\sum_{i=0}^{100}\sum_{j=1}^{2}w_{ij}^{s}m_{ij}^{y}.$$

In this formula, $m_{ij}^{y}=D_{ij}^{y}/P_{ij}^{y}$ represents the age- and sex-specific mortality rate for age *i* (*i* = 0,1,…,99,100+) and sex *j* (*j* = 1 for men and *j* = 2 for women) in year *y* ($D_{ij}^{y}$ being the number of deaths and $P_{ij}^{y}$ the size of the population for age *i* and sex *j* in year *y*), while $w_{ij}^{s}=P_{ij}^{s}/P^{s}$ is the proportion of people with age *i* and sex *j* in the reference year *s*, *P*^*s*^ being the total size of the population in that year. The dSMR for the reference year *y* = *s* = 2020 corresponds to the crude mortality rate in that year, so that *m*^2020^ = *D*^2020^/*P*^2020^, *D*^2020^ being the total number of deaths observed in year 2020.

In order to evaluate an excess mortality in 2020, we compared *m*^2020^ with *m*^*y*^ for various years *y* via a relative change in dSMR (expressed in %) calculated as follows:

$$100\frac{m^{2020}-m^{y}}{m^{y}}=100∙\frac{m^{2020}}{m^{y}}-100.$$

Although these quantities were obtained for the whole Swiss population, it is still important to calculate confidence intervals around them, as “the number of events that actually occurred may be considered as one of a large series of possible results that could have arisen under the same circumstances”, as underlined by Curtin and Klein [9]. A 95% confidence interval for a relative change can be obtained as follows [14, 15]:

$$100∙exp\left\{log\left(\frac{m^{2020}}{m^{y}}\right)\pm 1.96∙\sqrt{\frac{1}{D^{2020}}+\frac{\sum_{i=0}^{100}\sum_{j=1}^{2}{D_{ij}^{y}(\frac{P_{ij}^{2020}}{P_{ij}^{y}})}^{2}}{{\left(\sum_{i=0}^{100}\sum_{j=1}^{2}\frac{D_{ij}^{y}P_{ij}^{2020}}{P_{ij}^{y}}\right)}^{2}}}\right\}-100.$$

A relative change was considered statistically significant when the corresponding 95% confidence interval did not contain the value 0. Similar calculations were made for different sub-populations, such as men and women, or for selected age groups, although we could not consider finer groups than those available for the number of deaths in 2020 (5-year age groups with a last open class of 90+). To obtain smoother results that are not overly dependent on what may have happened in a given year, and to allow further comparison, we also carried out comparisons of the dSMR observed in 2020 with the dSMR obtained by pooling data from 2015–2019, as has been done in other studies [16, 17].

An alternative way to summarize mortality in a given year *y* and for a given sex *j* is to calculate life expectancy at birth $e_{0j}^{y}$ resulting from the age- and sex-specific mortality rates observed in that year. Applying a piecewise exponential model [18], we estimated life expectancy at birth as follows:

$$e_{0j}^{y}=\int_{x=0}^{110}\exp\left(-\sum_{i=0}^{x}{\bar{m}}_{ij}^{y}\right)dx.$$

In this formula, ${\bar{m}}_{ij}^{y}$ are mortality rates for age *i* (*i* = 0,⋯,110), sex *j* (*j* = 1,2) and year *y* (*y* = 1970,⋯,2020), calculated and assumed constant within the 5-years each age groups available for 2020 (last open class 90+). In words, $e_{0j}^{y}$ is obtained as the area under the survival function for sex *j* (*j* = 1,2) and year *y* (*y* = 1970,⋯,2020) estimated from these mortality rates. We validated our method by comparing the resulting life expectancies for the years 1970–2018 with those available in the HMD database, finding an average absolute difference of 0.07%, with no difference exceeding 0.3% (or even 0.08% since 2010). Life expectancies for the years 1876–1969 were taken directly from the HMD database. We were then able to compare $e_{0j}^{2020}$ with $e_{0j}^{y}$ for various years *y*, either via an absolute change in life expectancy (expressed in months), $12(e_{0j}^{2020}-e_{0j}^{y}),$ or via a relative change in life expectancy (expressed in %), $100(e_{0j}^{2020}-e_{0j}^{y})/e_{0j}^{y}$.

## Results

**Fig 1:**
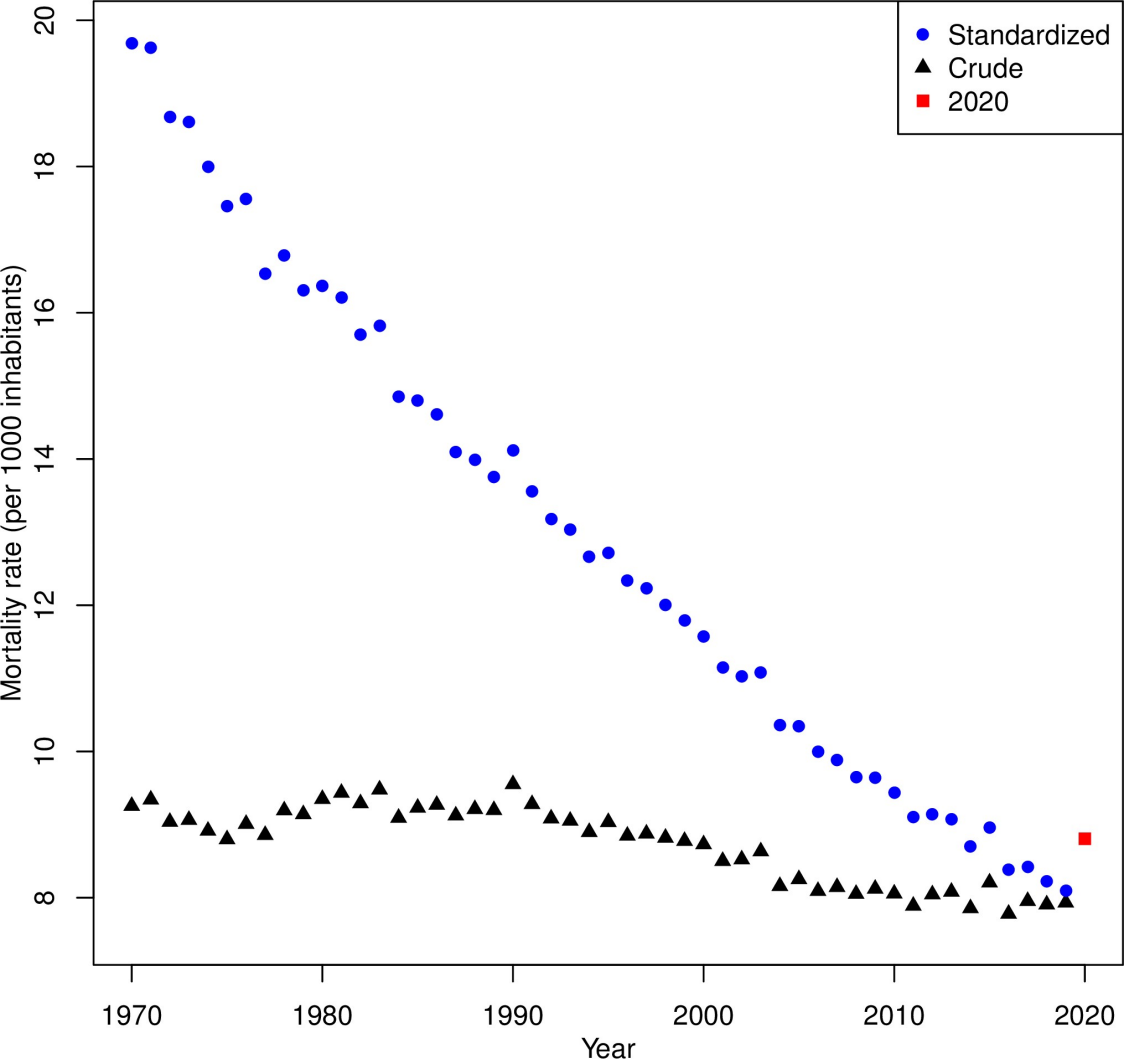
Crude and directly standardized mortality rates in Switzerland for the period 1970–2020 (data from the Swiss Federal Statistical Office and the Human Mortality Database).

Fig 1 shows the crude and standardized mortality rates in Switzerland calculated for the years 1970–2020. Plotting crude and standardized rates over 50 years allowed us to illustrate the very different trends over time observed for these two indicators. In contrast to the crude mortality rates, which have remained roughly constant, the standardized mortality rates have decreased by a factor of about 2.5 over the last 50 years, reflecting the continuing and impressive reduction in mortality. In 2020, however, both crude and standardized mortality rates were higher than in previous years. The crude mortality rate in 2020 returned to the level of about 20 years before. Because of the absence of a clear trend, it remained close to (and sometimes even higher than) rates observed in the early 1970s. In contrast, the standardized mortality rate in 2020 returned to the level of only 5–6 years before, being lower than in 2015 and any year prior to and including 2013. Similar conclusions were drawn when calculations were done separately for men and women (S1 Fig).

Table 1 presents the relative changes in dSMR when comparing the year 2020 with the years 2019, 2015 and 2010, and with pooled years 2015–2019. One can see that the observed mortality in 2020 increased by 8.8% compared to 2019, this relative difference being greater for men (10.6%) than for women (7.2%). In absolute numbers, this represents an increase of 8007 deaths (6143 after standardization) compared to 2019. Mortality has increased by 4.8% compared to years 2015–2019, while it has decreased by 1.7% compared to 2015, and by 6.7% compared to 2010.

**Table 1:** Summary of changes in directly standardized mortality rates (dSMR) and in life expectancy comparing 2020 to previous years.

	dSMR			Life Expectancy					
*Change in 2020 vs*:	Total	Men	Women	Total		Men		Women	
	%	%	%	%	Months	%	Months	%	Months
**2019**	**+8.8**	+10.6	+7.2	**-0.7**	**-7.5**	-1.0	-9.7	-0.5	-5.3
**2015–2019**	**+4.8**	+5.8	+3.8	**-0.3**	**-3.1**	-0.4	-4.1	-0.2	-2.0
**2015**	**-1.7**	-1.5	-1.9	**+0.4**	**+3.7**	+0.5	+4.6	+0.3	+2.8
**2010**	**-6.7**	-8.2	-5.2	**+1.2**	**+11.4**	+1.3	+12.9	+1.0	+9.9

**Fig 2:**
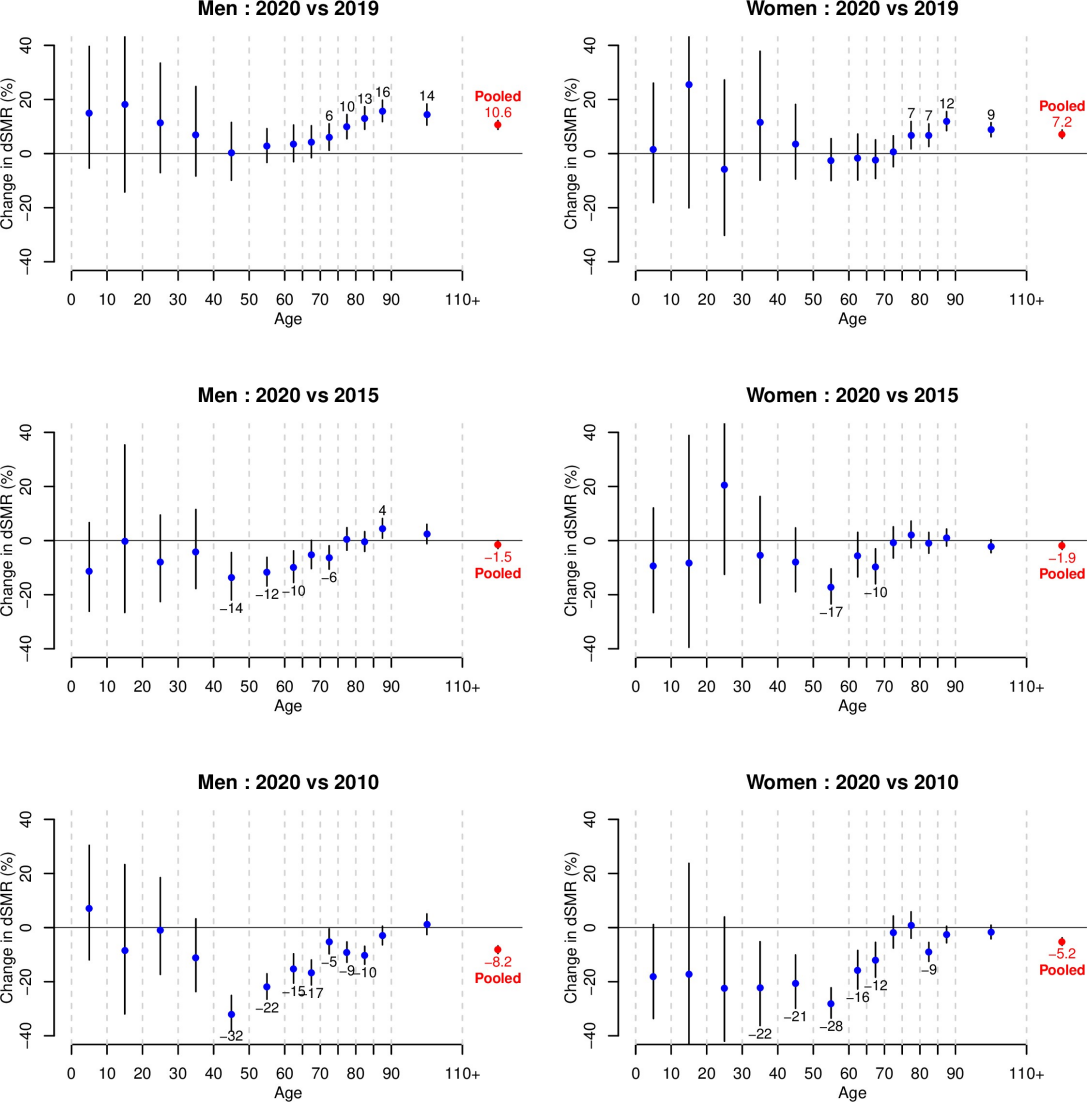
Relative change in directly standardized mortality rates (dSMR) when comparing 2020 with 2019, 2015 and 2010 in selected age groups, separately for men and women, as well as for pooled age classes, together with 95% confidence intervals. Estimated changes (expressed in %) are provided when statistically significant.

Fig 2 shows the relative change in dSMR in 2020 compared to years 2019, 2015 and 2010, separately for specific age groups, and for men and women, together with 95% confidence intervals. One can see that the dSMR increased significantly in 2020 compared to 2019 for all age groups over 70 years for men, respectively for all age groups over 75 years for women. For younger age groups, we found no significant change in dSMR. Compared to 2015, the dSMR increased significantly in only one age group for men (85–90), and in no age group for women, while it decreased significantly in 4 age groups for men (between 40 and 75), and in 2 age groups for women (50–60 and 65–70). Compared to 2010, the dSMR decreased significantly in most age groups for both men and women, showing that mortality in Switzerland was lower in 2020 than in 2010 at all ages, including in the oldest age groups.

**Fig 3:**
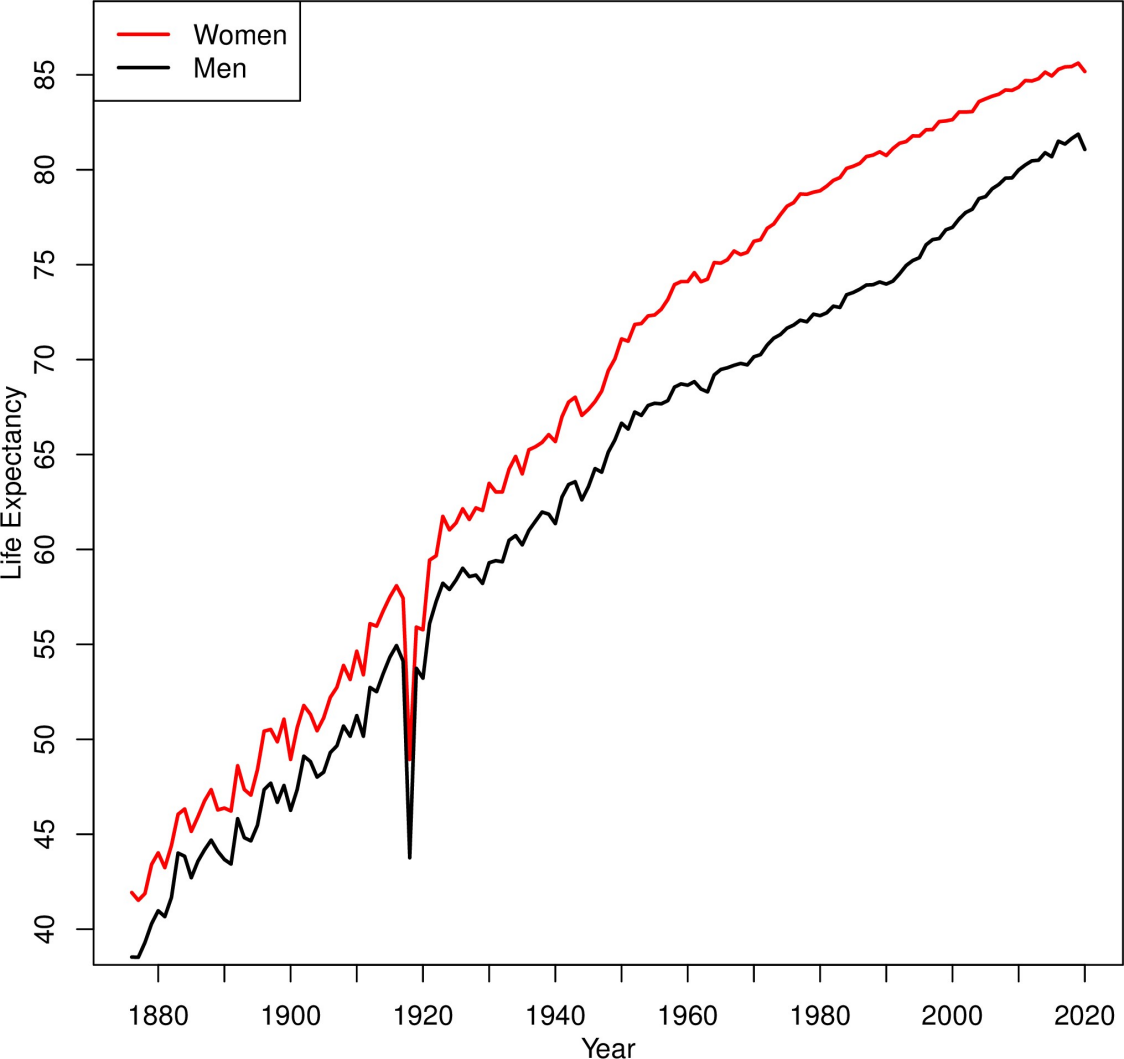
Life expectancy in Switzerland for the period 1876–2020 for men and women (data from the Swiss Federal Statistical Office and the Human Mortality Database).

Fig 3 shows life expectancy at birth in Switzerland for the years 1876–2020, separately for men and for women. The secular increase of life expectancy is spectacular, rising from about 40 years in 1876, to 81.9 years for men and 85.6 years for women in 2019. Also spectacular is the sudden fall in life expectancy in 1918 following the Spanish Flu, where men lost 10.4 years and women 8.5 years compared to 1917. In 2020, life expectancy has also decreased compared to 2019, although the decrease was much lower, with men reaching 81.1 years and women 85.2 years in 2020. Thus, the decrease was 9.7 months (or 1.0%) for men and 5.3 months (or 0.5%) for women. Averaged over both sexes, the decrease was 7.5 months (or 0.7%). As for dSMR, life expectancy in Switzerland in 2020 returned to the level of about 5 years before, being higher in 2020 than in any year prior to and including 2015. See Table 1 for a comparison of life expectancy in 2020 with other years.

## Discussion

In this paper, we compared mortality in Switzerland in 2020 with that of previous years. We found that the standardized mortality rate was 8.8% higher in 2020 than in 2019. This increase was larger for men than for women and was only statistically significant for the oldest age groups (over 70 years for men and over 75 years for women). The fact that the oldest age groups were the most affected explains why the decrease in life expectancy at birth in 2020 compared to 2019 was much smaller (0.7%) than the increase in standardized mortality rate. According to both indicators, mortality in Switzerland in 2020 corresponds to a return to the level observed 5–6 years before, i.e., around 2015.

Standardization is a well-recognized technique in the field of mortality, making it possible to analyze the evolution of mortality over time, adjusting for changes in the age structure of the population. In fact, while the evolution of crude mortality rates is a mixture of two opposing trends (reduction in mortality and aging of the population), standardized mortality rates show clearly that mortality in 2020 returned to the level of 2015. Of course, the analysis of changes in standardized mortality rates does not entirely substitute the detailed analysis of changes in mortality rates by age [19, 20]. For example, when age-specific rates vary in opposite directions in different age groups, any summary measure, such as the standardized rate ratio, will mask these differences by providing a summary in which positive and negative variations in age-specific rates offset each other. This is why we also performed comparisons in selected age groups in order to identify the ages primarily affected by changes in mortality. Ahmad et al. [5], using a similar method based on standardized and age-specific mortality rates, found for the United States a 15.9% increase of the standardized mortality rate in 2020 compared to 2019, an excess mortality almost twice as large as our estimation for Switzerland. However, they also reported mortality rates that increased sharply with age and were higher in men than in women.

In contrast to a standardized mortality rate, which is affected by mortality at all ages more or less equally, life expectancy at birth is a summary indicator of mortality that gives much more weight to mortality at a young age than at an advanced age. It is a popular indicator which is often used to compare mortality across countries or periods. The loss of life expectancy observed in Switzerland in 2020 (9.7 months for men and 5.3 months for women) is the greatest since 1944 for men (where the loss was 11.5 months), and since 1962 for women (where the loss was 5.8 months). In particular, it is greater than the loss observed in 2015, a year with a severe flu in winter and a heat wave in summer, responsible for a loss of 2.5 months for each sex.

Our estimates of the loss of life expectancy in Switzerland in 2020 are close to those obtained by Aburto et al. [8], who reported a loss of almost 12 months for men, and slightly more than 6 months for women. Of note, these results were calculated using five broad age classes (0–14, 15–64, 65–74, 75–84, and 85+). Using the same five age classes, we would have calculated a loss of 11.9 months for men and 6.6 months for women, almost perfectly matching their results. Heuveline and Tzen [7] calculated a loss in life expectancy in Switzerland in 2020 of 1.07 years, which is higher than our estimates. However, their methodology differed from ours as it was based on COVID-19 mortality and not all-cause mortality. The fact that the number of deaths attributed to COVID-19 in Switzerland surpassed the excess all-cause mortality found for 2020 (see below) may partly explain the difference between their estimate and ours.

On the other hand, our estimated loss in life expectancy in Switzerland in 2020 is smaller than the loss of 1.1 years (13.6 months), respectively 1.26 years (15.1 months), estimated in the United States by Andrasafay and Goldman [6], respectively Heuveline and Tzen [7], and of 1.2 years (14.4 months) for men and 0.9 years (10.8 months) for women estimated in England and Wales by Aburto et al. [3] (the latter based on the first 47 weeks of 2020 only). These results are consistent with the fact that the United States and the United Kingdom are among the countries that suffered the most from COVID-19 in 2020, reaching respectively the 8th and 11th highest mortality rate, with Switzerland ranked 21st. All these losses, however, are still much lower than those observed in 1918, the year of the Spanish Flu, when life expectancy decreased by more than 10 years for men and more than 8 years for women in Switzerland. Unlike COVID-19, the Spanish Flu killed young adults between 20 and 40 years of age much more than the elderly.

Just as the massive increase in mortality observed in 1918 was mainly attributed to Spanish Flu, any excess mortality observed in 2020 will inevitably be interpreted as consequences (direct and indirect) of the COVID-19 pandemic, unquestionably the major event in 2020. In fact, the number of deaths attributed to COVID-19 in 2020 (about 7400) was higher than the number of all excess deaths observed in 2020 with respect to 2019 (6143). Of note, the situation was different in other countries. In the United States, deaths attributed to COVID-19 accounted for only about 75% of all excess deaths in 2020 [4, 21]. One possible explanation could be underreporting of COVID-19 deaths in the United States, e.g. due to a lack of testing, although many other factors could simultaneously come into play in different directions. For example, an excess of all-cause mortality greater than the mortality attributed to COVID-19 could also be explained by indirect effects of COVID-19 on mortality, or by a disruption of the medical system, whereas the opposite could be explained by overreporting of COVID-19 deaths due to multimorbidity or harvesting effects. In a preprint, Kelly [17] found that the excess all-cause mortality in 2020 was greater than the mortality attributed to COVID-19 in about half of the 36 countries she considered (including the United States), whereas the reverse was true for the other half (including Switzerland). Examination of the impact of COVID-19 waves on excess mortality could be further explored using time series data.

As mentioned in the introduction, our analysis is not definitive because it takes some time before complete mortality data are available. This analysis is therefore intended to be repeated in a few months or years. In particular, it is possible that the mortality reported in 2020 will increase slightly in the coming months, as some deaths might be reported with delay. We recognize that this is a limitation of our study. Nevertheless, having followed the weekly updates of the FSO figures since the very beginning of 2021, we expect that our results are not far from having converged. In fact, according to the internet site quoted above, no new deaths for 2020 have been reported since April 6. Extending the analysis beyond the year 2020, once the pandemic will be over, is another topic for future work.

Although it is somewhat too early to draw definitive conclusions, there is no doubt that there was an excess mortality in Switzerland in 2020, linked to the COVID-19 pandemic and due to increased mortality among the elderly. However, it is also clear that the loss of life expectancy generated by this excess mortality was less than one year, bringing the mortality level back to 2015. Of course, these results occurred in the presence of important protection measures taken in 2020 by the government to fight the COVID-19 pandemic and it is very difficult to estimate what would have happened without them. These measures, by reducing the number of contacts among people, certainly limited the spread of the virus, and thus hospital overcrowding and mortality. On the other hand, the social and economic costs of these measures are also undeniable and there will be a lively debate to take stock of the actions undertaken. We believe that the loss of life expectancy observed in the pandemic year 2020, which we estimated at a few months, will be an important element in this debate.

## Supporting information

S1 Fig Crude and directly standardized mortality rates for men and women in Switzerland for the period 1970–2020 (data from the Swiss Federal Statistical Office and the Human Mortality Database). (TIF)

## References

[1] Beaney T, Clarke J, Jain V, Golestaneh A, Lyons G, Salman D, et al. (2020). Excess mortality: the gold standard in measuring the impact of COVID-19 worldwide? Journal of the Royal Society of Medicine, 113, 329–334. doi: 10.1177/0141076820956802 32910871 PMC7488823

[2] World Health Organization (2016). World Health Statistics 2016: Monitoring health for the SDGs Annex B: tables of health statistics by country, WHO region and globally. https://www.who.int/gho/publications/world_health_statistics/2016/Annex_B/en/.

[3] Aburto J, Kashyap R, Schöley J, Angus C, Ermisch J, Mills MC, et al. (2021). Estimating the burden of the COVID-19 pandemic on mortality, life expectancy and lifespan inequalities in England and Wales: A population-level analysis. Journal of Epidemiology and Community Health. Epub ahead of print. 10.1136/jech-2020-215505 PMC7818788 33468602

[4] Woolf S, Chapman D, Salbo R, Weinberger D, Hill L (2021). Excess deaths from COVID-19 and other causes in the US, March 1, 2020, to January 2, 2021. JAMA. Epub ahead of print. 10.1001/jama.2021.5199 PMC8019132 33797550

[5] Ahmad F, Cisewski J, Minino A, Anderson R (2021). Provisional mortality data–United States, 2020. Morbidity and Mortality Weekly Report, 70, 519–522. doi: 10.15585/mmwr.mm7014e1 33830988 PMC8030985

[6] Andrasafay T, Goldman N (2021). Reduction in 2020 US life expectancy due to COVID-19 and the disproportionate impact on the Black and Latino populations. PNAS, 118, e2014746118. doi: 10.1073/pnas.2014746118 33446511 PMC7865122

[7] Heuveline P, Tzen M (2021). Beyond deaths per capita: comparative COVID-19 mortality indicators. BMJ Open, 11, e042934. doi: 10.1136/bmjopen-2020-042934 33692179 PMC7948156

[8] Aburto J, Schöley J, Kashnitzky I, Zhang L, Rahal C, Missov T, et al. (2021). Quantifying impacts of the COVID-19 pandemic through life expectancy losses: a population-level study of 29 countries. medRxiv. 10.1093/ije/dyab207 PMC8500096 34564730

[9] Curtin L, Klein R (1995). Direct standardization (age-adjusted death rates). Healthy People 2000 Statistical Notes, 6, 1–10. 11762384

[10] Neison F (1844). On a method recently proposed for conducting inquiries into the comparative sanatory condition of various districts. Journal of the Royal Statistical Society of London (now the Royal Statistical Society), 7, 40–68.

[11] Spiegelman M, Marks H (1966). Empirical testing of standards for the age adjustment of death rates by the direct method. Human Biology, 38, 280–292. 5977532

[12] Miettienen O (1972) Components of the crude risk ratio. American Journal of Epidemiology, 96, 168–172. doi: 10.1093/oxfordjournals.aje.a121443 4261806

[13] Fleiss JL, Levin B, Cho Paik M (2003). Statistical methods for rates and proportions. Wiley Series in Probability and Statistics Eds.

[14] Breslow N and Day N (1987). Statistical methods in cancer research, Volume II–The design and analysis of cohort studies. London: Oxford University Press. 3329634

[15] Fay MP (1999). Approximate confidence intervals for rate ratios from directly standardized rates with sparse data. Communications in Statistics–Theory and Methods, 28, 2141–2160.

[16] Modig K, Ahlbom A, Ebeling M (2021). Excess mortality from COVID-19: weekly excess death rates by age and sex for Sweden and its most affected region. The European Journal of Public Health, 31, 17–22. doi: 10.1093/eurpub/ckaa218 33169145 PMC7717265

[17] Kelly G (2021). Covid-19 and excess mortality rates not comparable across countries. medRxiv. 10.1017/S0950268821001850 PMC8365039 34338184

[18] Friedman M (1982). Piecewise exponential models for survival data with covariates. Annals of Statistics, 10, 101–113.

[19] Woolsey T (1959). Adjusted death rates and other indices of mortality. Chapter 4 in Linder F.E. and Grove R.D. (Eds.), Vital statistics rates in the United States, 1900–1940. Washington, D.C.: U.S. Government Printing Office.

[20] Elveback L (1966). Discussion of “Indices mortality and tests of their statistical significance.” Human Biology, 38, 322–324.

[21] Rossen L, Branum A, Ahmad F, Sutton P, Anderson R (2021). Update on excess deaths associated with the COVID-19 pandemic–United States, January 26, 2020-February 27, 2021. Morbidity and Mortality Weekly. 10.15585/mmwr.mm7015a4 PMC8344999 33857065

