# Supplementary figures and images for: A first analysis of excess mortality in Switzerland in 2020
Source: PLoS One. 2021 Jun 17;16(6):e0253505. doi: 10.1371/journal.pone.0253505 (PMC8211252; doi:10.1371/journal.pone.0253505)

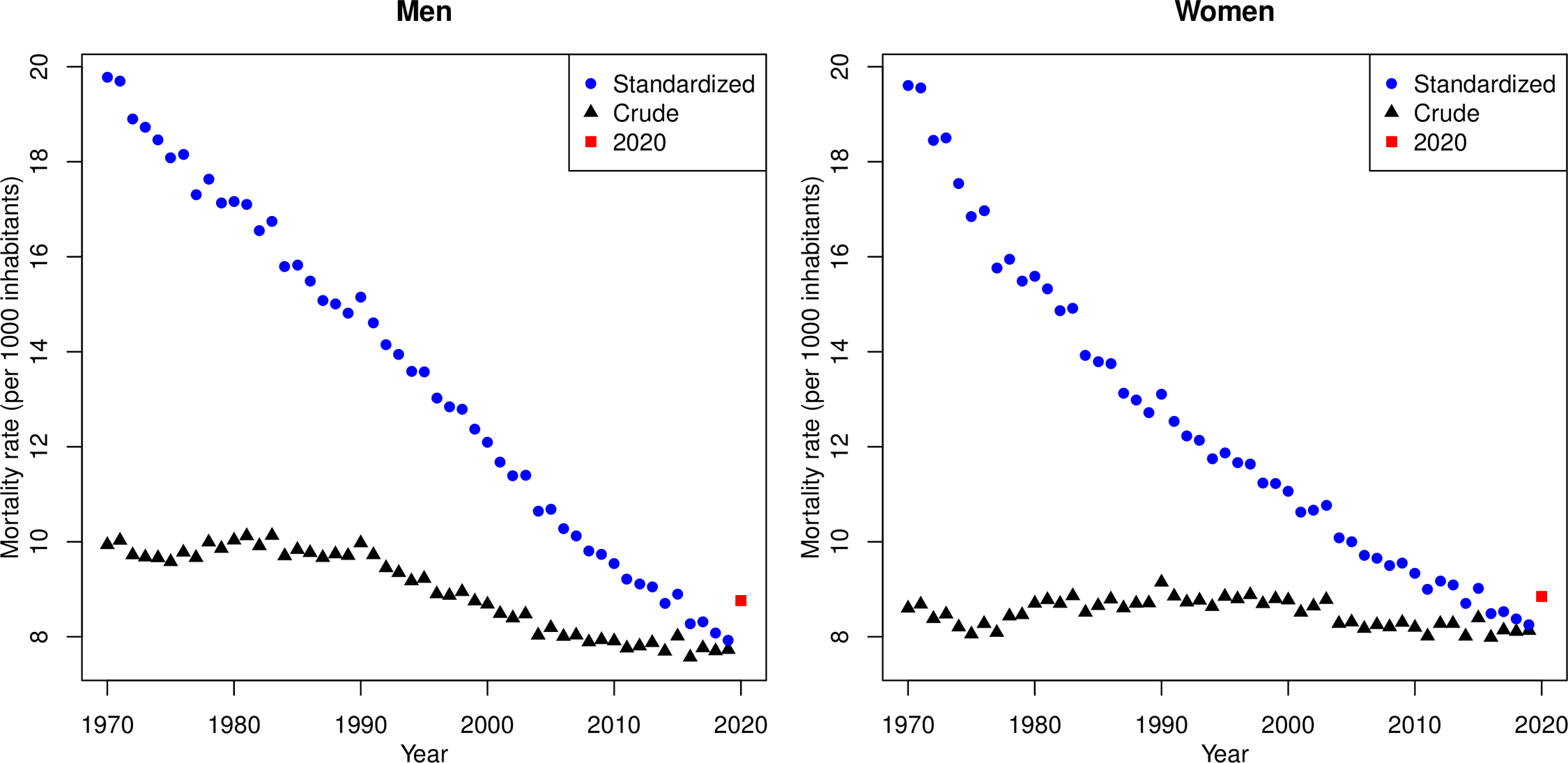

Supplement: S1 Fig — (TIF) [file pone.0253505.s001.tif]
